# Study of the fundamental frequency in elderly women with hearing loss

**DOI:** 10.1016/S1808-8694(15)30082-3

**Published:** 2015-10-19

**Authors:** Giovana dos Santos Baraldi, Lais Castro de Almeida, Lucila Leal Calais, Alda Cristina de Carvalho Borges, Ingrid Gielow, Mauricio Raymundo De Cunto

**Affiliations:** a Master’s degree in science, UNIFESP. Speech therapist; b Master’s degree in science, Sao Paulo Federal University - Paulista Medical School. Speech therapist; c Master’s degree in science, Sao Paulo Federal University - Paulista Medical School. Speech therapist; d Doctor in Communication Disorders, UNIFESP. Adjunct professor at UNIFESP/ Paulista Medical School; e Doctor in Communication Disorders, UNIFESP/Paulista Medical School. Professor at UNIFESP/Paulista Medical School; f Graduate student (master’s degree) in biosafety, USP - Sao Carlos. Electronic engineer. Sao Paulo Federal University

**Keywords:** elderly, hearing loss, voice

## Abstract

Increased life expectancy raises demands for special attention for the elderly population; speech, language and hearing science deals with their communication disorders. Hearing loss is a common disorder affecting this age group. It is known that the auditory feedback system is essential to human vocalizing, as it organizes voice production. **Aim:** To assess and correlate the hearing system and the Fundamental Frequency (F0) of women who have variable degrees of sensorineural hearing loss. **Material and Method:** a cross-sectional descriptive study. 30 women with a mean age of 75.95 (SD = 7,41) were included. Inclusion criteria were: symmetric sensorineural hearing loss, a high-frequency sloping configuration, and a type A tympanogram. Subjects underwent Pure Tone Audiometry, a Word Recognition Test, Tympanometry, and Voice Assessment. **Results:** Patients with higher degrees of hearing loss showed an increased fundamental frequency. **Conclusion:** In aged individuals with hearing loss, audiovocal monitoring is altered, resulting in voice parameter changes.

## INTRODUCTION

Brazilian demography has changed significantly in past decades according to data from the Brazilian Institute of Geography and Statistics.^1^ In 2003 the life expectancy at birth for both genders reached 71.3 years, an increase of 0.8 years compared to 2000 (70.5 years). This change in Brazilian demography has led to an increase in the number of elderly people and their specific health needs. In this scenario we have seen the development of speech therapy for the elderly and frequent studies on hearing and voice. Presbycusis, or age-related hearing impairment, is sensorineural hearing loss with a gradual sloping configuration.^2^ Presbyphonia is the aging voice, which includes voice tremor, increased intensity, and a soprous and harsh quality of voice.^3^ Hearing and voice operate jointly in communication, where hearing monitors voice production. Monitoring one’s own voice is essential for good communication; the auditory system is able to regulate voice parameters such as intensity, extension and frequency. One of the most frequently studied voice parameters in the literature is the fundamental frequency (F_0_). F_0_ is physiologically determined by the number of cycles that vocal folds produce in a second, or the number of repeated glottal cycles.^4^ The mean F_0_ for adult women (aged between 18 and 45 years) that speak Brazilian Portuguese is 204 Hz. The F_0_ decreases with age; in women over 45 years it decreases to about 191 Hz.^5^ As age progresses, changes of the F_0_ are related not only to anatomical and physiological alterations of the larynx, but also to the difficulty of monitoring voice as a result of age-related hearing loss.

The aim of this paper was to assess and to correlate hearing and voice F_0_ values in elderly patients with varying degrees of auditory sensitivity.

## MATERIAL AND METHODS

This study was analyzed and approved by the Research Ethics Committee of the Sao Paulo Federal University for research on human beings under the protocol number CEP 1189/04 of the National Health Council. The study sample included 30 female subjects aged between 60 and 93 years (mean 76.23 years, standard deviation = 8.17) referred from the Geriatrics and Gerontology Institute of the Sao Paulo Federal University (UNIFESP). The evaluation of hearing and voice was done between June 2004 and May 2005. Subjects underwent basic assessments of hearing and voice in an acoustic booth as follows:

- pure tone audiometry: measuring of air conduction audiometry thresholds at 250 to 8000 Hz and bone conduction auditory thresholds at 500 to 4000 Hz in both ears. The descending method was used for measuring thresholds with both transducers, using audible and inaudible sound, as proposed by Carhart & Jerger.^6^ This procedure includes the following steps: a) the sound stimulus is presented at an easily perceived intensity; b) intensity is reduced at 10 dB intervals until the stimulus becomes inaudible; c) intensity is increased at 5 dB intervals until the subjects once again perceives sound. The audibility threshold is considered as the lowest intensity level at which subjects responds to two of four presentations according to the steps described above.
- voice audiometry: investigation of the speech recognition threshold (SRT) and the speech recognition percentage rate (SRPR). The SRT was obtained by a descending procedure using disyllables presented directly. Subjects were asked to repeat the speech stimulus. The lowest intensity level at which subjects recognized 50% of the stimuli - two of four words presented - was defined as the speech recognition threshold. The SRPR, a supraliminar test to establish the listener’s ability to recognize speech stimuli under ideal listening conditions, was investigated using an initial intensity 40 dB over tone threshold means at 500, 1000 and 2000 Hz. If patients reported discomfort, the intensity was reduced in 10 dB steps until the patient reported the best intensity for obtaining the percentage rate of speech recognition. Stimuli were presented orally. The list of 25 phonetically balanced monosyllables we used was proposed by Pen and Mangabeira.^7^
- acoustic immitance testing: tympanometry and investigation of contralateral acoustic reflections, done to discard middle ear involvement. Tympanometry permits inferences on the mobility of the tympanic and ossicular systems; acoustic reflection thresholds correspond to the lowest intensity that can alter static compliance.

Audiometries were classified according to two criteria, as follows:

1. Silman, Silverman^8^: classification of the degree of sloping losses using mean frequencies of 500, 1000 and 2000 Hz, and of 2000, 3000 and 4000 Hz. A degree of loss was given for low frequencies (500Hz, 1 and 2 KHZ) and another for high frequencies (2, 3 and 4 KHZ).
2. Lloyd, Kaplan^9^: classification of the sloping audiometric configuration according to threshold differences between octaves; descending configurations are classified as flat, gradual, sloping and abrupt.

Subjects were instructed to issue the vowel /a/ naturally, at usual intensity and frequency, in a prolonged and sustained manner, and to count the numbers 1 to 10 (sample of connected speech). Voices were recorded in an acoustic booth where maximum noise was 60dB; subjects were seated during recordings. A preamplifier, a VHS stereo HI-FI system and an Audio 20 Plantronic microphone were used for voice recording, as recommended by De Cunto and Menezes (2004).10 Voice samples were digitized at a sample frequency of 32000 Hz using the Sound Forge software, version 4.5. Acoustic analysis was done using the CSL software from Kay Elemetrics Corporation, version 1993/1994, module MVDP, from which the fundamental frequency was extracted.

After auditory and voice assessment, data was analyzed statistically using the Kruskal-Wallis, Mann-Whitney, chi-square and equality of two proportions non-parametric tests. The significance level was 0.10 (10%).

## RESULTS

Starting with pure tone audiometry, right and left ear results were compared to check for symmetry between ears, as published papers have suggested that the left ear performs better in presbyacusis subjects.^12^ There was no statistically significant difference between ears in this study; ears were therefore analyzed jointly. As a result of this there were 30 ears for 30 subjects in our analysis.

## DISCUSSION

Hearing and voice systems change with age. The acuity and sensitivity of hearing, and the accuracy, speed, resistance and coordination of voice all decrease with aging. These changes go hand in hand with other bodily changes, and vary from person to person and according to each organism and lifestyle. Studies on the influence of hearing on voice production in the elderly are not frequent in the literature; there have been few studies correlating both systems. There have been reports from authors such as those by Shindo and Hanson^12^ showing that hearing loss and dependency in the elderly exacerbate the impact of dysphonia. Notwithstanding the lack of studies on hearing and voice production, auditory methods have been successfully employed in the rehabilitation of dysphonia, based on the principle that improved hearing positively affect voice production.13 Based on this principle, it is easy to imagine how the hearing and voice systems are interrelated, and how adaptation of an individual hearing aid in an hearing-impaired elderly patient can help improve auditory sensitivity and voice production, reduce voice tension and intensity, and prevent damage to the phonatory system.

The fundamental frequency, one of the most studied voice production parameters, may be directly related to the auditory monitoring capability. There are studies showing that when auditory monitoring is altered, the F_0_ voice parameter also changes.^14^ Table 1 shows the distribution of degrees of hearing loss at low and high frequencies, and the distribution of audiometric configurations. Moderate loss predominated at low frequencies (30%), followed by normal hearing (28.3%) and mild loss (26.7%). The similar analysis applied to high frequencies shows that moderate loss predominated (33,3%), followed by moderate-severe loss (25%) and severe loss (11.7%). These findings are similar to those of Katsarkas and Ayukawa, which investigated 68 subjects aged over 50 years; most of these subjects had normal hearing or mild hearing loss at low frequencies, and 37% of subjects had moderate and severe loss at high frequencies.^11^ Other papers have suggested that elderly subjects have increased high frequency thresholds, particularly over 3KHz,^15^, ^16^, ^17^ showing that frequency loss is more significant at higher frequencies. Studies on elderly subjects commonly report a lower auditory threshold at high frequencies in elderly patients.

**Table 1:** Distribution of auditory sensitivity degrees and the audiometric configuration for low and high frequencies for both ears. (n=60).

	Gender	female	
		n	%
500Hz, 1 and 2 KHz	normal	23	28.3%
	mild	16	26.7%
	moderate	18	30%
	moderate-severe	6	10%
	severe	3	5%
	total	60	100%
2, 3 and 4 KHz	normal	11	18.3%
	mild	7	11.7%
	moderate	20	33.3%
	moderate-severe	15	25%
	severe	7	11.7%
	total	60	100%
audiometric configuration	flat	9	15%
	gradual	28	46.7%
	abrupt	17	28.3%
	Rampa	6	10%

	total	60	100%

The classification of audiometric configurations was also used in parallel with the degree of severity. The gradual configuration (41.3%) predominated, followed by the abrupt configuration (33.,8%), the flat configuration (12.5%) and the sloping configuration (12.5%). These results suggest that the most recurring configuration in the elderly was the gradually sloping type, in which there is an auditory threshold difference between audiogram octaves of more than 5 to 12 dB. Other authors that investigated the same age group have reported that most of the subjects had a downward sloping audiometric configuration where losses occur over 2kHz.18 A study of 331 elderly subjects of both genders also showed that a sloping audiometric configuration was more frequent.^19^

Table 2 shows audiometric findings in detail, where the correlation between the F_0_ of sustained issuing of vowel “a”, the sample of connected speech and the degree of hearing loss at low frequencies may be seen. The mean F_0_for subjects with mild hearing loss (144.44Hz), moderate hearing loss (160.30Hz), moderate-severe hearing loss (188.23Hz) and severe hearing loss (201.27Hz) leads to the conclusion that the F_0_ value after sustained issuing of the vowel “a” was significantly increased at low frequency hearing loss. No significant difference was found in the comparison of F_0_ values of the connected speech sample and low frequency degrees. Table 3 shows the correlation between the same F_0_ values and high frequencies, where a significant difference was found for both vowel “a” and connected speech. Mean F_0_ values for sustained issuing of the vowel “a” and the connected speech sample were as follows: 137.22 and 154.32 Hz (mild hearing loss), 154.23 and 189.63 Hz (moderate hearing loss), 174.37 and 183.31 Hz (moderate-severe hearing loss), and 170.02 and 234.16 Hz (severe hearing loss). We noted that F_0_ is significantly increased in severe high frequency hearing loss both for the sustained vowel and the connected speech sample. There are few published papers relating the F_0_ voice pattern to hearing loss. Weatherley et al. analyzed fundamental frequency measurements in 19 normal-hearing subjects and compared them with measurements in 21 subjects with hearing loss. The mean F_0_ value for these subjects (all aged over 60 years) was 189.68 Hz for women with hearing loss; no difference was seen between subjects with hearing loss and normal-hearing subjects.20 An increased F_0_ in severe hearing loss may be explained by studies on auditory monitoring that report increased F_0_ values with reduced auditory monitoring.14 Subject in our study all had hearing loss, so it may be assumed that increased mean F_0_ values were due to decreased auditoryvoice monitoring.

**Table 2:** Correlation of F_0_ values for sustained issuing of vowel “a” and the sample of connected speech with the degree of loss at 500 Hz, 1 and 2KHz.

500 Hz, 1 and 2KHz	mean	standard deviation	minimum	maximum	p-value	
	normal	163.82	39.44	68.0	207.9	0.001*
	mild	144.44	29.04	85.8	192.3	
F_0_ /a/ (Hz)	moderate	160.30	33.57	85.8	213.5	
	moderate-severe	188.23	5.32	182.4	196.8	
	severe	201.27	10.63	195.1	213.5	
	normal	187.65	34.95	138.2	269.7	0.104
	mild	185.45	37.94	137.9	250.3	
F_0_ speech (Hz)	moderate	187.15	23.65	148.8	250.3	
	moderate-severe	181.97	23.61	151.6	200.2	
	severe	258.78	27.05	227.5	274.4	

**Key:** F0 = fundamental frequency

**Table 3:** Correlation of F_0_ values for sustained issuing of vowel “a” and the sample of connected speech with the degree of loss at 2, 3 and 4KHz.

2, 3 e 4 KHz	mean	standard deviation	minimum	maximum	p-value	
	normal	169.43	34.33	68.0	187.4	0.004*
	mild	137.22	41.39	68.0	207.9	
F_0_ /a/ (Hz)	moderate	154.23	21.62	90.2	192.3	
	moderate-severe	174.37	29.07	85.8	207.9	
	severe	170.02	56.61	85.8	213.5	
	normal	193.87	40.31	138.2	269.7	0.001*
	mild	154.32	19.40	137.9	183.8	
F_0_ speech (Hz)	moderate	189.63	21.91	143.4	235.1	
	moderate-severe	183.31	29.19	148.8	250.3	
	severe	234.16	34.36	189.3	274.4	

F_0_ values were also correlated with audiometric configurations. Table 4 shows the correlation between the F_0_ of sustained issuing of vowel “a”, the connected speech sample and audiometric configurations. We noted a statistically significant difference between the connected speech sample F_0_ and audiometric configurations. The mean F_0_value was low for the abrupt configuration (176.32 Hz) compared to the flat configuration (191.63 Hz), the gradual configuration (197.61 Hz) and the sloping configuration (189.85 HZ). The conclusion is that the F_0_ mean was higher in the gradually sloping audiometric configuration, in which there is a 5 to 12 dB difference in auditory thresholds between frequency octaves.

**Table 4:** Correlation of F_0_ values for sustained issuing of vowel “a” and the sample of connected speech and audiometric configuration.

configuration	mean	standard deviation	minimum	maximum	p-value	
	abrupt	147.78	47.86	68.0	213.5	0.147
F_0_ /a/ (Hz)	gradual	169.73	22.47	132.4	213.5	
	flat	179.71	7.56	167.3	187.4	
	sloping	138.72	42.30	90.2	184.7	
	abrupt	176.32	38.08	137.9	250.3	0.052*
F_0_ speech (Hz)	gradual	197.61	35.75	143.4	274.4	
	flat	191.63	30.30	173.0	269.7	
	sloping	189.85	5.69	183.8	196.5	

Our data suggest the auditory feedback regulates voice production by monitoring different voice parameters. In the elderly it is clear how hearing loss and voice changes are related. Hearing affects voice production by changing its parameters, especially in subjects with marked hearing loss. These indicators point to the need for early auditory rehabilitation and routine assessments of voice disorders in the elderly. Early auditory rehabilitation in elderly patients may help reduce voice changes, directly affecting communication and social interaction.

## CONCLUSION

The fundamental frequency (F_0_) of a sustained vowel was increased in marked low and high frequency hearing loss. The fundamental frequency (F_0_) of the connected speech sample was increased in the sloping audiometric configuration where marked loss was seen over 2 KHz.
